# Tumor-Derived circRNAs as Circulating Biomarkers for Breast Cancer

**DOI:** 10.3389/fphar.2022.811856

**Published:** 2022-02-15

**Authors:** Yunhe Yu, Wenfang Zheng, Changle Ji, Xuehui Wang, Mingkuan Chen, Kaiyao Hua, Xiaochong Deng, Lin Fang

**Affiliations:** ^1^ Department of Breast and Thyroid Surgery, Shanghai Tenth People’s Hospital, School of Medicine, Tongji University, Shanghai, China; ^2^ School of Medicine, Tongji University, Shanghai, China

**Keywords:** breast cancer, circRNA, biomarkers, diagnostic model, liquid biopsy

## Abstract

Early diagnosis is the key to improving the prognosis of breast cancer (BC) patients; however, there are currently no circulating biomarkers that demonstrate good sensitivity and specificity. This study applied circular RNA (circRNA) microarray analysis, screening, and verification in BC plasma samples to identify three tumor-derived differentially expressed circRNAs: hsa_circ_0000091, hsa_circ_0067772, and hsa_circ_0000512. We constructed a diagnostic model using logistic regression analysis in the training set and established an optimal diagnostic model based on the three circRNAs, which showed sensitivity, specificity, and area under the curve (AUC) values of .971, .902, and .974, respectively. We then verified the diagnostic model in the test set which showed satisfactory stability for BC diagnosis. Additionally, the expression of hsa_circ_0000091 in plasma correlated with axillary lymph node (ALN) metastasis, TNM stage, and prognosis of BC patients. Furthermore, hsa_circ_0000091 combined with ultrasound showed predictive ability for ALN metastasis, with an AUC of .808. These findings suggested that the three identified circRNAs can be used as circulating biomarkers for BC diagnosis, with hsa_circ_0000091 potentially representing a prognostic biomarker for BC and novel approach for predicting ALN metastasis.

## Introduction

Breast cancer (BC) is a complex malignant tumor that shows the highest morbidity among women worldwide (Siegel et al., 2020), with diagnosis at an early stage key to improving BC patient prognosis. Although numerous methods for the early detection of BC have been proposed, imaging techniques, including ultrasound and mammography, are the main screening and diagnostic approaches (Bevers et al., 2018). Carcinoembryonic antigen (CEA), carbohydrate antigen 125 (CA125), carbohydrate antigen 15-3 (CA15-3), and carbohydrate antigen 19-9 (CA19-9), which are considered traditional circulating biomarkers for BC, are used less frequently for the early diagnosis of BC because of their low sensitivity and specificity (Wang et al., 2017; Li et al., 2019a; Nam et al., 2019; Li et al., 2020). Hence, there is lack of available biomarkers for BC liquid biopsy, and identifying those showing both specificity and sensitivity continues to be challenging. Therefore, an optimized set of circulating biomarker molecules and effective algorithms is needed to develop an accurate liquid biopsy method for BC.

Circular RNA (circRNA) is a type of noncoding RNA produced by back-splicing. Unlike linear RNAs, circRNAs are circular in structure and do not possess 5′ caps or 3′ tails, making them highly stable. Additionally, circRNAs play critical roles in various cancers (Yu et al., 2019) and are both abundant and stable in bodily fluids (Li et al., 2015; Zuo et al., 2020; Wang et al., 2021). Several studies report that circulating circRNAs can serve as diagnostic biomarkers for various cancers (Li et al., 2019b; Wang et al., 2021), including hepatocellular carcinoma (Wu et al., 2020a; Sun et al., 2020), gastric cancer (Tang et al., 2018), and chronic lymphocytic leukemia (Wu et al., 2020b). Nevertheless, the potential of circulating circRNAs as biomarkers for diagnosing BC remains largely unknown.

Tumor cells can secrete RNAs, including circRNAs, into the circulatory system, and these secreted RNAs are closely related to tumor proliferation and metastasis (Tkach and Théry, 2016). Thus, we hypothesized that tumor-derived circRNAs present in circulation can help diagnose cancers with improved specificity and sensitivity. In this study, we determined the value of tumor-derived circRNAs in plasma for BC diagnosis and developed an optimal diagnostic model for BC based on a combination of three tumor-derived plasma circRNAs.

## Materials and Methods

### Patients and Samples

The inclusion criteria for the BC group were as follows: 1) BC confirmed by pathological report, 2) patients having not undergone preoperative radiotherapy and/or chemotherapy, and 3) absence of other malignant tumors or serious chronic diseases. BC patients who underwent surgery at the Shanghai Tenth People’s Hospital and met the aforementioned criteria between 2017 and 2020 were enrolled in this study. The inclusion criterion for the normal group was healthy individuals without any benign tumors, malignant tumors, or serious chronic diseases.

Venous blood samples were collected from all participants, with preoperative blood samples collected before surgery for BC patients (BC group) and normal plasma samples collected from healthy individuals (normal group). Postoperative blood samples were collected 3 days after BC surgery, and metastatic blood samples were collected after confirmation metastasis. All blood samples were centrifuged at 3,000 rpm for 10 min at 4°C. Following high-speed centrifugation at 12,000 ×*g* for 10 min, the plasma was separated and stored at −80°C. A total of 523 blood samples (202 preoperative samples, 202 normal samples, 102 postoperative samples, and 17 metastatic samples) were included in this study. BC preoperative and normal plasma samples were randomized into training and test sets. This study was approved by the Institutional Ethics Committee of Shanghai Tenth People’s Hospital, and the study methodology met the criteria outlined in the Declaration of Helsinki.

### CircRNA Microarray

We used the human circRNA array (Arraystar, Rockville, MD, United States ) for analyses of five BC tissues and matched adjacent normal tissues. Quantification of total RNA extracted from each tissue sample was performed using a NanoDrop ND-1000 (Thermo Fisher Scientific, Waltham, MA, United States ), with sample preparation and microarray hybridization performed according to the manufacturer’s instructions.

### RNA Isolation and Quantitative Reverse Transcription Polymerase Chain Reaction

Total RNA in plasma was extracted using an EZ-press Serum/Plasma RNA purification kit (Yingze, Suzhou, China) according to the manufacturer’s instructions. cDNA was synthesized using the HiScript III RT SuperMix kit (Vazyme Biotech, Nanjing, China), and qRT-PCR was conducted using SYBR Green Master Mix (Yeasen, Shanghai, China). Primer sequences were designed and synthesized by RiboBio (Guangzhou, China), with the 18S gene sequence used as an internal reference for circRNAs. The primer sequences were as follows: hsa_circ_0000091 forward, 5′-CAG​CTG​TTT​ACC​AGA​GTG​CAT​GA-3′ and reverse, 5′-CGA​TGC​GTT​TTC​TAA​TCT​GGT​TC-3′; hsa_circ_0067772 forward, 5′-TGC​CAG​CAG​TTC​TGA​CAT​T-3′ and reverse, 5′-TCT​TTG​GGT​ACT​CCC​TCT​T-3′; hsa_circ_0000512 forward, 5′-TTT​GCC​GGA​GCT​TGG​AAC-3′ and reverse, 5′-ATC​TCC​TGC​CCA​GTC​TGA​CC-3′; and 18S forward, 5′-TAG​AGG​GAC​AAG​TGG​CGT​TC-3′ and reverse, 5′-CGC​TGA​GCC​AGT​CAG​TGT-3′. The relative expression of circRNAs was assessed using the 2^−ΔΔCT^ method.

### Statistical Analysis

Comparisons between paired specimens were analyzed using the Wilcoxon matched-pairs signed rank test, whereas the Mann–Whitney *U* test was used for unpaired samples. Comparisons between circRNA expression and patient clinical features were conducted using the chi-squared test. The receiver operating characteristic (ROC) curve and corresponding area under the curve (AUC) were determined for individual and combined circRNAs. Disease-free survival (DFS) was defined as the time interval from the date of surgery to the date of recurrence or final contact. The Kaplan–Meier method was applied for survival analysis. Log-rank tests were used to determine statistical significance. Statistical analysis was performed using SPSS (v.25.0; IBM Corp. Armonk, NY, United States ) and GraphPad Prism (v.8.0; GraphPad Software, La Jolla, CA, United States ). Data are presented as the mean ± standard deviation (SD) and were considered statistically significant at *p* < .05.

## Results

### Screening for Candidate Plasma circRNAs

**FIGURE 1:**
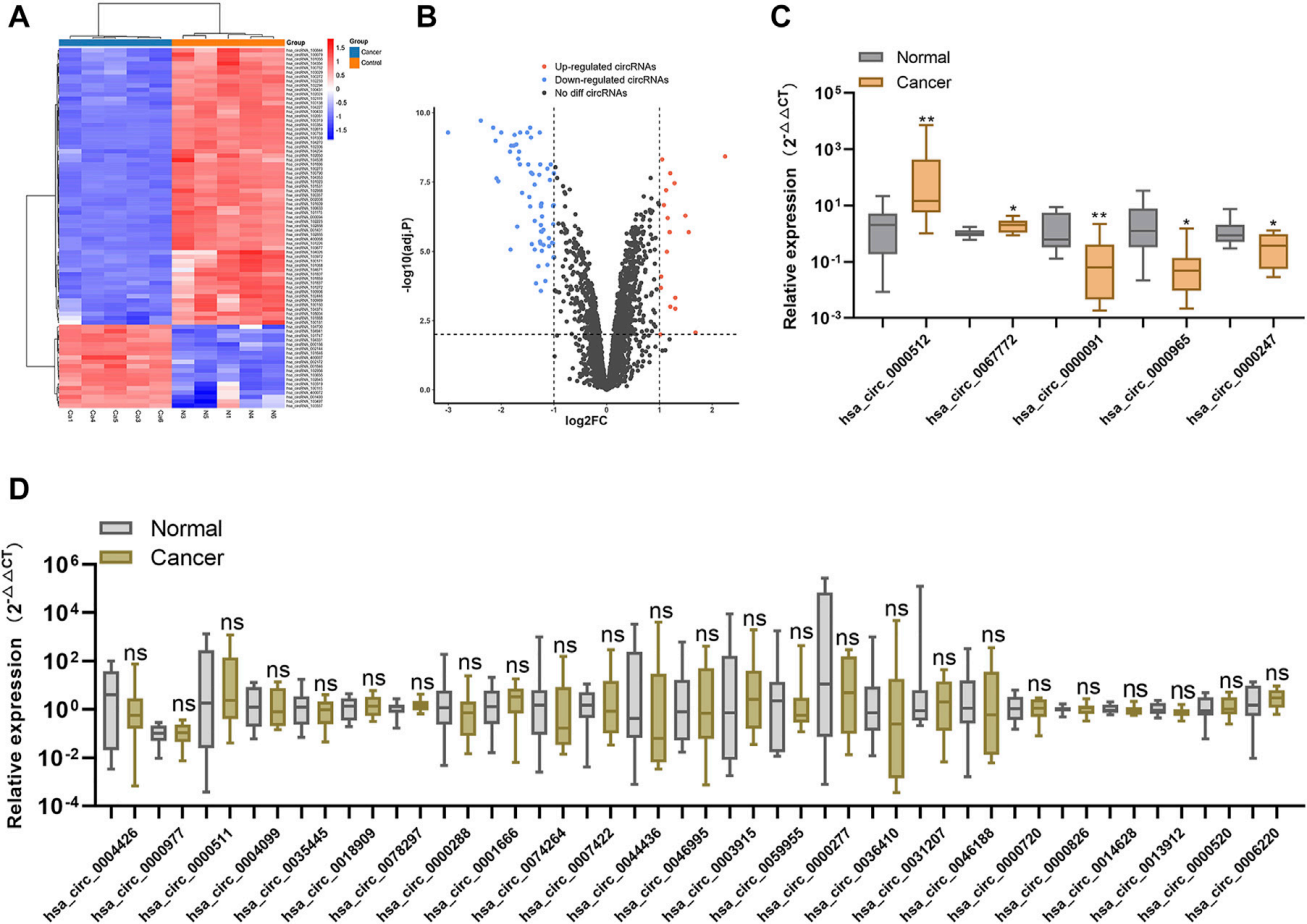
Screening for candidate plasma circRNAs **(A)**. Heat map showing the 82 differentially expressed circRNAs between five paired human BC tissues and normal tissues **(B)**. Volcano plot showing significantly downregulated and upregulated circRNAs between BC and normal tissues **(C,D)**. Relative expression levels of the 30 circRNAs showing the highest degree of differential expression between 10 BC plasma samples and normal plasma samples according to qRT-PCR. *p < .05, **p < .01, ***p < .001, and ****p < .0001. Normal, normal plasma samples; ns, no significance.

We performed high-throughput human circRNA microarray analysis using samples from five BC tissues and matched adjacent normal tissues to detect circRNA expression. The identification criteria for differentially expressed circRNAs were absolute fold change (|FC|) ≥2 and *p* < .01, and the data were used to construct heat maps and volcano plots to visualize the results. We identified 82 differentially expressed circRNAs, including 19 upregulated and 63 downregulated circRNAs, between BC tissues and normal adjacent tissues (Figures 1A,B).

To screen candidate tumor-derived circRNAs in plasma, we analyzed the expression of the top 30 differentially expressed circRNAs (ranked by |FC|) in 10 BC plasma samples versus 10 normal plasma samples using qRT-PCR. We identified five circRNAs (hsa_circ_0000091, hsa_circ_0000512, hsa_circ_0067772, hsa_circ_0000247, and hsa_circ_0000965) as differentially expressed in BC plasma samples relative to normal plasma samples (Figures 1C,D), resulting in their selection as candidate plasma circRNAs.

### Establishment of the Diagnostic Model in the Training Set

**FIGURE 2:**
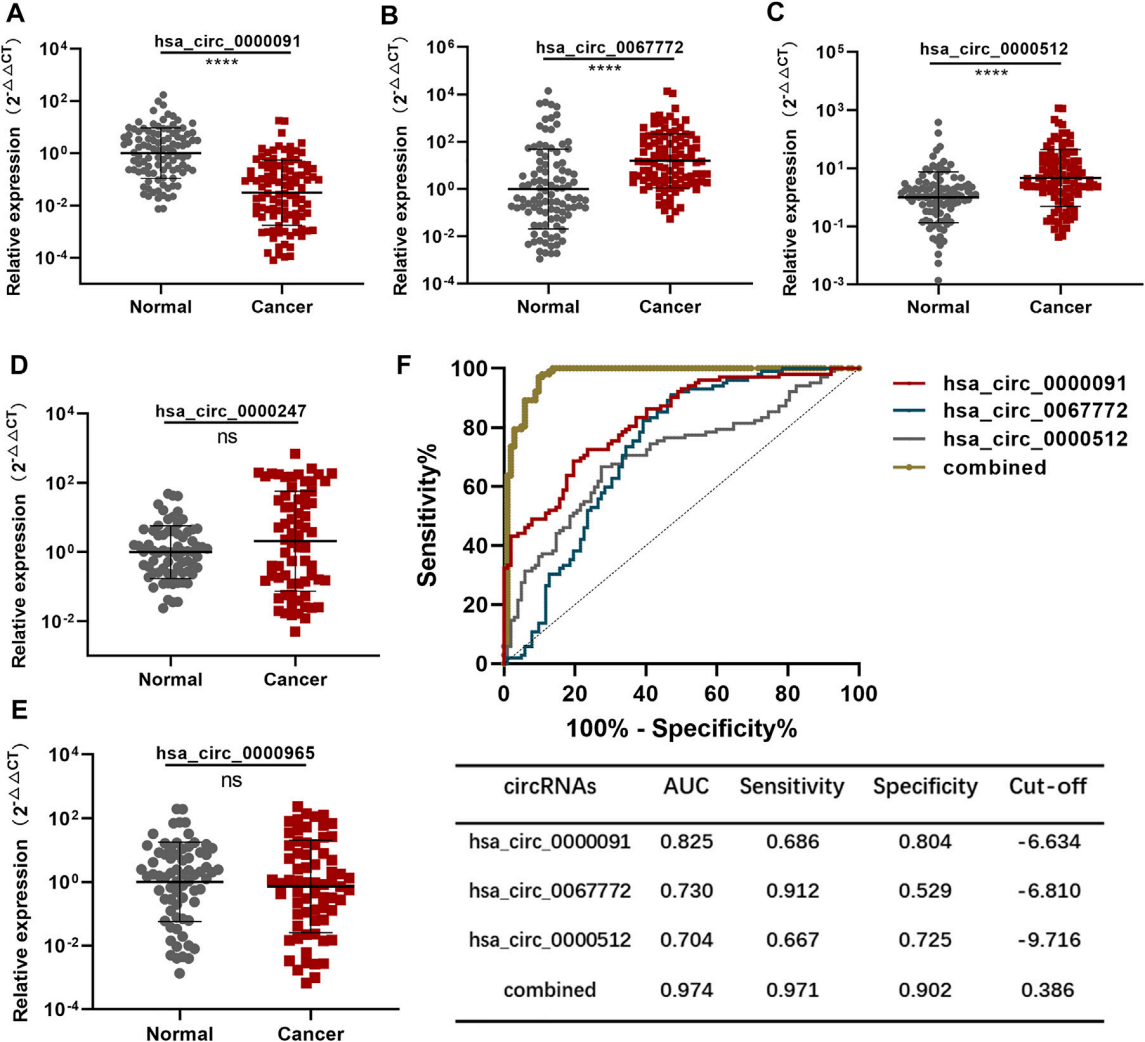
Establishment of the diagnostic model using the training set **(A–C)**. Detection of hsa_circ_0000091, hsa_circ_0067772, and hsa_circ_0000512 expression by qRT-PCR in 102 normal plasma samples vs. 102 BC plasma samples included in the training set **(D,E)**. Detection of hsa_circ_0000247 and hsa_circ_0000965 expression by qRT-PCR in 70 normal plasma samples vs. 70 BC plasma samples **(F)**. ROC curves for the three individual three circRNAs and their combined use generated using the training set. Data are represented as the mean ± SD. *p < .05, **p < .01, ***p < .001, and ****p < .0001. Normal, normal plasma samples; combined, the combination of the three circRNAs; ns, no significance.

We expanded the number of BC and normal plasma samples in each group to 102 and considered this the training set. QRT-PCR results revealed that hsa_circ_0000091 expression decreased, whereas expression of hsa_circ_0067772 and hsa_circ_0000512 increased in BC plasma samples relative to that in normal plasma samples (Figures 2A–C). However, the expression of hsa_circ_0000247 and hsa_circ_0000965 showed no significant difference with increased sample size (Figures 2D,E); therefore, we used hsa_circ_0000091, hsa_circ_0067772, and hsa_circ_0000512 to establish a diagnostic model.

We applied the ROC curve to the training set to evaluate the sensitivity and specificity of the circRNAs for BC diagnosis. Separate analysis of individual circRNAs and the three circRNAs combined revealed AUCs for hsa_circ_0000091, hsa_circ_0067772, and hsa_circ_0000512 of .825 [95% confidence interval (CI): .769–.880], .730 (95% CI: .659–.801), and .704 (95% CI: .632–.776), respectively. To establish the diagnostic model, we performed logistic regression of the three circRNAs using the following equation:

Logit(P)=5.640+0.132* hsa_circ_0067772+1.195* hsa_circ_0000512−1.035* hsa_circ_0000091.

As expected, the AUC of the combination of the three circRNAs was as high as .974 (95% CI: .952–.996) along with significantly improved sensitivity and specificity (Figure 2F). Therefore, we established the diagnostic model using the combination of three plasma circRNAs, which showed satisfactory diagnostic capacity for BC.

### Identification of the Three Tumor-Derived circRNAs

**FIGURE 3:**
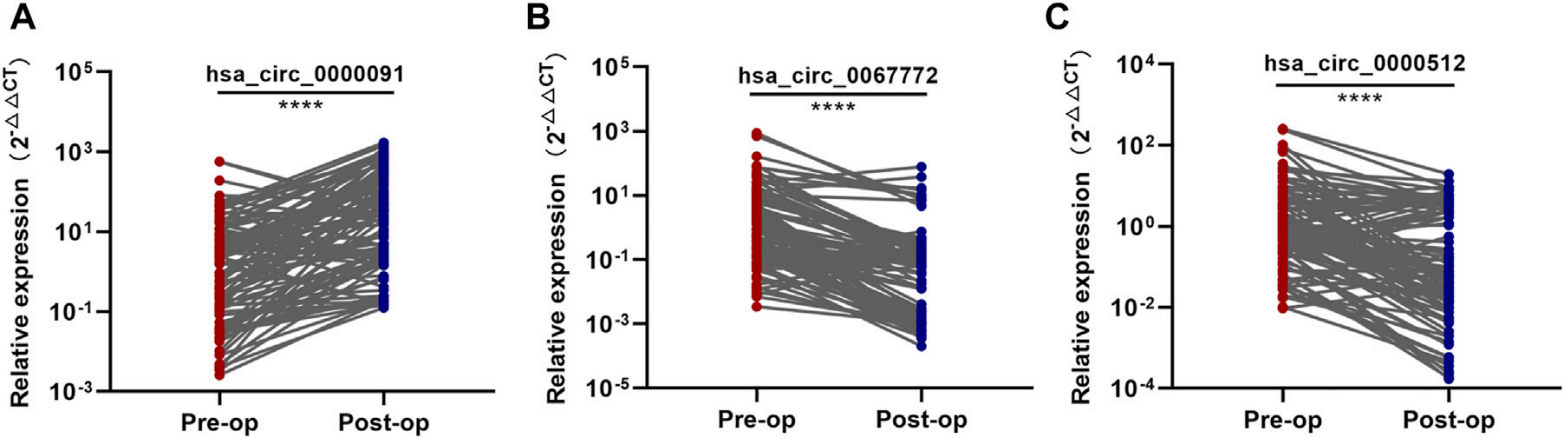
Identification of the three tumor-derived circRNAs **(A–C)**. Comparison of the expression levels of hsa_circ_0000091, hsa_circ_0067772, and hsa_circ_0000512 between pre and postoperative samples from 102 BC patients included in the training set. *p < .05, **p < .01, ***p < .001, and ****p < .0001. Pre-op, preoperative plasma samples; Post-op, postoperative plasma samples.

To confirm that these three circRNAs were breast tumor–derived circRNAs, we detected their expression in postoperative plasma samples from the 102 BC patients enrolled in the training set using qRT-PCR. After resection of the breast tumors, we found that hsa_circ_0000091 expression was significantly higher and hsa_circ_0067772 and hsa_circ_0000512 expression was significantly lower in postoperative plasma samples than in preoperative plasma samples (Figures 3A–C), suggesting similar expression profiles for these three circRNAs following removal of the breast tumors to those in normal individuals. These results identified hsa_circ_0000091, hsa_circ_0067772, and hsa_circ_0000512 as tumor-derived plasma circRNAs.

### Validation of the Diagnostic Model

**FIGURE 4:**
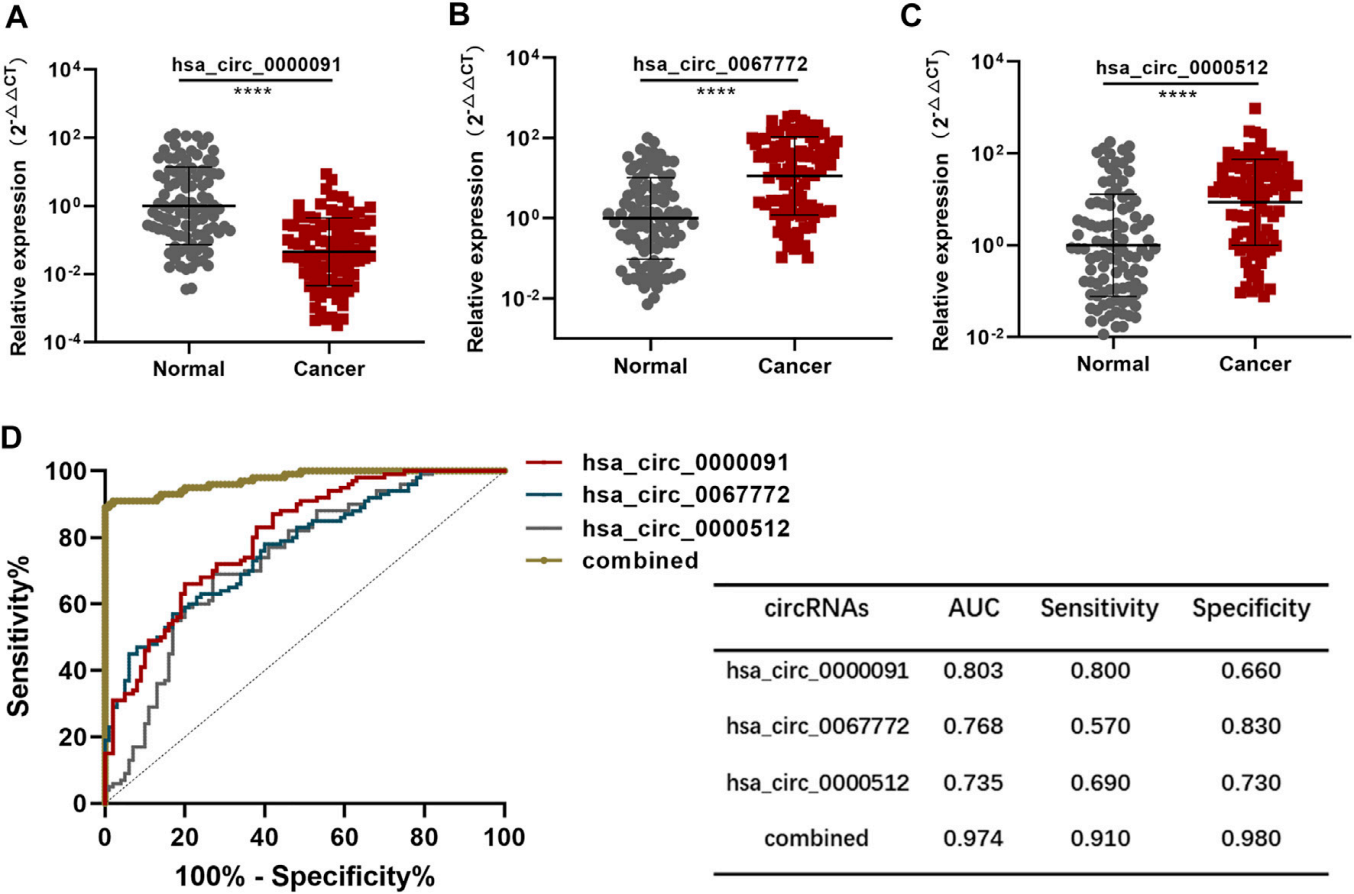
Validation of the diagnostic model **(A–C)**. Detection of hsa_circ_0000091, hsa_circ_0067772, and hsa_circ_0000512 expression by qRT-PCR of 100 normal plasma samples vs. 100 BC plasma samples included in the test set **(D)**. ROC curves for the three individual three circRNAs and their combined use generated using the test set. *p < .05, **p < .01, ***p < .001, and ****p < .0001. Normal, normal plasma samples; combined, the combination of the three circRNAs.

We used a total of 100 BC plasma samples and 100 normal plasma samples as the test set. Analysis using the chi-squared test revealed no difference in the features of patients enrolled between the training and test sets (Supplementary Table S1). Moreover, the expression of hsa_circ_0000091, hsa_circ_0067772, and hsa_circ_0000512 in the test set showed the same trends as that in the training set (Figures 4A–C). Based on the diagnostic model established using the training set, the AUC of the diagnostic model in the test set was .974 (95% CI: .955–.993) (Figure 4D), indicating that the diagnostic model was stable.

### Comparison of the Identified circRNAs With Traditional Biomarkers

Levels of traditional biomarkers, including CEA, CA125, CA15-3, and CA19-9, above the reference range are considered to indicate positive diagnosis of BC. Here, we identified a demarcation point for distinguishing high or low expression of circRNAs according to the ROC curve generated from the training set. Following calculation of the Youden index, we determined the cutoff value for circRNA expression as the maximum of the Youden index (Figure 2F), with the cutoff values for the circRNAs then used as discriminators. As shown in Table 1, we found that both separate and combined use of the identified plasma circRNAs showed a higher positivity rate for BC diagnosis than traditional biomarkers in both the training and test sets.

**TABLE 1:** Number of positive and negative samples in each set.

Events	Discrimination in the		Discrimination in the	
	training set (*n* = 102)		test set (*n* = 100)	
	Positive	Negative	Positive	Negative
Hsa_circ_0000091	75 (73.53%)	27	83 (83.00%)	17
Hsa_circ_0067772	84 (82.35%)	18	62 (62.00%)	38
Hsa_circ_0000512	68 (66.67%)	34	89 (89.00%)	11
Combination of circRNAs	82 (80.39%)	20	70 (69.00%)	30
CEA	17 (16.67%)	85	14 (14.00%)	86
CA125	14 (13.73%)	88	12 (12.00%)	88
CA15-3	20 (19.61%)	82	18 (18.00%)	82
CA19-9	29 (28.43%)	73	25 (25.00%)	75
Combination of traditional biomarkers	48 (47.06%)	54	43 (43.00%)	57

### Hsa_circ_0000091 Expression Correlates With the Prognosis of BC Patients

**FIGURE 5:**
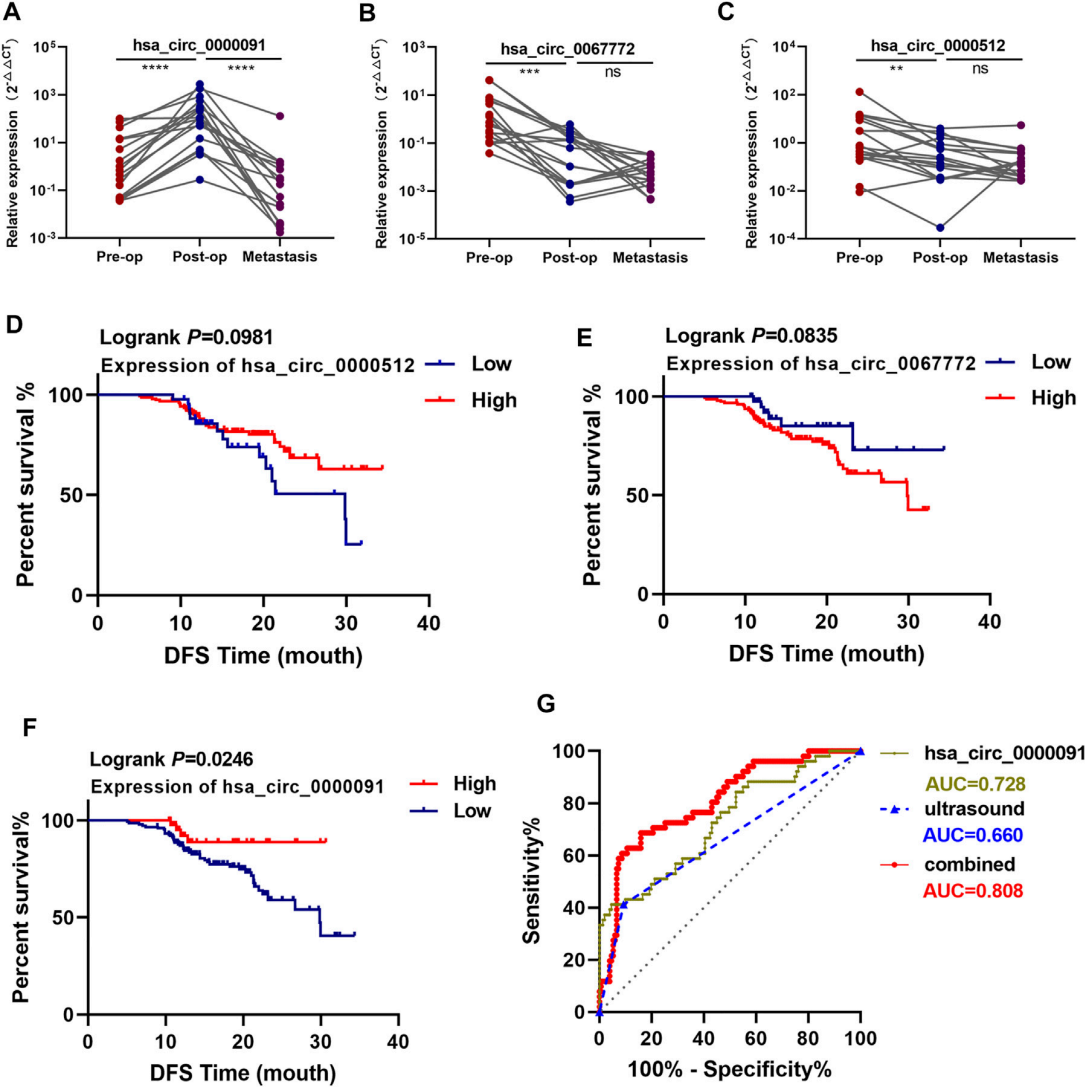
Hsa_circ_0000091 expression correlates with the prognosis of BC patients. **(A–C)**. Comparison of the expression of hsa_circ_0000091, hsa_circ_0067772, and hsa_circ_0000512 among preoperative, postoperative, and metastatic plasma samples from 17 patients with metastatic BC. **(D–F)**. Survival analysis of BC patients according to hsa_circ_0067772, hsa_circ_0000512, and hsa_circ_0000091 expression as detected by qRT-PCR (*n* = 202). The demarcation points for levels of the three circRNAs used as cutoff values. Log-rank tests were used to determine statistical significance **(G)**. ROC curves for the use of hsa_circ_0000091 alone or combined with ultrasound to predict ALN metastasis. *p < .05, **p < .01, ***p < .001, and ****p < .0001. Pre-op, preoperative plasma samples; Post-op, postoperative plasma samples; metastasis, metastatic plasma samples; combined, the combination of hsa_circ_0000091 and ultrasound; ns, no significance.

We collected 17 plasma samples from patients with metastatic tumors during the course of postoperative follow-up and examined the expression of hsa_circ_0000091, hsa_circ_0067772, and hsa_circ_0000512. We found that the relative expression of hsa_circ_0000091 in metastatic plasma samples decreased compared with that observed in postoperative plasma samples, whereas no significant difference was observed in hsa_circ_0067772 and hsa_circ_0000512 expression between postoperative and metastatic plasma samples (Figures 5A–C). Moreover, the low expression of hsa_circ_0000091 correlated with poor prognosis of BC patients (Figure 5F), whereas we observed no difference in prognostic efficacy between hsa_circ_0067772 and hsa_circ_0000512 for BC patients (Figure 5D,E).

Using the identified cutoff values as demarcation points, we found that hsa_circ_0000091 expression positively correlated with axillary lymph node (ALN) metastasis and TNM stage in 202 samples from BC patients (total of both the training and test sets) (Table 2), with an AUC of .728 (95% CI: .645–.811) for predicting ALN metastasis (Figure 5G). Furthermore, when combined with ultrasound, the ability of hsa_circ_0000091 expression to predict ALN metastasis improved [AUC: .808 (95% CI: .739–.877)] (Figure 5G).

**TABLE 2:** Relationships between circRNA expression and clinical features of BC patients.

Features	Total	hsa_circ_0000091		*p*	hsa_circ_0067772		*p*	hsa_circ_0000512		*p*
	(*n* = 202)	Low	High		Low	High		Low	High	
Age										
<50	44	32	12	.661	12	32	.273	8	36	.46
≥50	158	120	38		31	127		37	121	
Grades										
I	24	16	8	.389	5	19	.703	6	18	.903
II	95	70	25		18	77		20	75	
III	83	66	17		20	63		19	64	
T stage										
Tis	21	13	8	.344	6	15	.577	4	17	.789
T1	86	67	19		16	70		20	66	
T2	92	69	23		21	71		21	71	
T3	3	3	0		0	3		0	3	
ALN status										
ALN-	151	107	44	.013*	32	119	.955	30	121	.157
ALN+	51	45	6		11	40		15	36	
Stage										
I–II	165	119	46	.030*	37	128	.404	38	127	.587
III	37	33	4		6	31		7	30	
Molecular subtype										
Luminal A	46	35	11	.901	8	38	.397	11	35	.277
Luminal B	105	80	25		25	80		19	86	
HER2 overexpression	26	18	8		3	23		6	20	
TNBC	25	19	6		7	18		9	16	

The TNM staging system is based on tumor size (T), extent of the spread to the lymph nodes (N), and presence of metastasis (M).

*The bold values in Table 2 mean that p < 0.05, considered statistically significant.

### Target microRNA Prediction and Biological Pathway Enrichment Analysis

**FIGURE 6:**
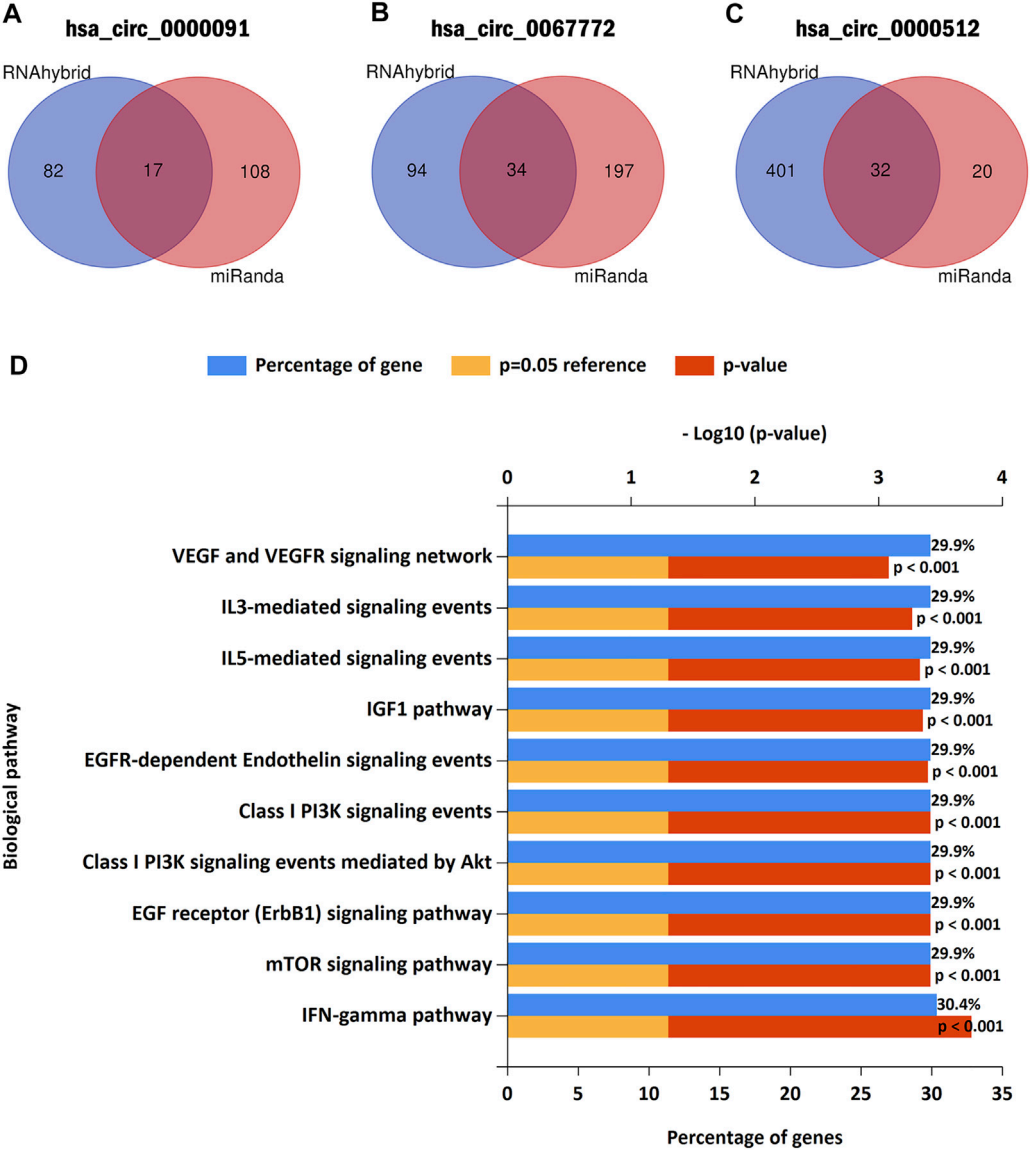
Predicted target miRNAs and biological pathway enrichment analysis **(A–C)**. Hsa_circ_0000091, hsa_circ_0067772, and hsa_circ_0000512 were predicted as sponges of 17, 34, and 32 miRNAs, respectively **(D)**. Biological pathway enrichment analysis indicated that hsa_circ_0000091, hsa_circ_0067772, and hsa_circ_0000512 participated in a variety of important signaling pathways.

To determine the role of the three circRNAs in BC regulation, we used miRanda and RNAhybird (http://bibiserv.techfak.uni-bielefeld.de/rnahybrid/) to predict the potential miRNA targets of the circRNAs. Integration of the data into a Venn diagram and selection of miRNAs from the intersections of the two databases identified 17, 34, and 32 miRNAs possibly targeted by hsa_circ_0000091, hsa_circ_0067772, and hsa_circ_0000512 for miRNA sponging, respectively (Figures 6A–C). Because different circRNAs can sponge the same miRNA, we obtained a total of 82 miRNAs after excluding duplicates (Supplementary Table S2). We then used the FunRich tool (http://www.funrich.org/) to perform biological pathway enrichment analysis for these 82 miRNAs. The results showed that hsa_circ_0000091, hsa_circ_0067772, and hsa_circ_0000512 are involved in 40 biological signaling pathways (Supplementary Table S3), including some important pathways that are strongly associated with cancer, such as the vascular endothelial growth factor (VEGF)/VEGF receptor, phosphoinositide 3-kinase/Akt, and interleukin 3 (IL-3)–mediated signaling events (Figure 6D). These findings suggest that hsa_circ_0000091, hsa_circ_0067772, and hsa_circ_0000512 regulate several miRNAs and are involved in a variety of important signaling pathways.

## Discussion

BC is a highly heterogenous malignant tumor that requires different treatment strategies according to different molecular subtypes, with patient prognosis varying immensely according to the molecular subtype (Liang et al., 2020); however, early diagnosis is critical for BC treatment. To date, early diagnosis of BC has mainly relied on imaging techniques among various diagnostic platforms, and liquid biopsy for BC remains in the nascent stage of use. Many studies confirmed the stability of circRNAs in the blood and that their expression can be measured in human blood accordingly, resulting in their use as circulating biomarkers for cancer diagnosis. For example, serum circFoxO3a can serve as a prognostic biomarker for squamous cervical cancer (Tang et al., 2020). Additionally, a combination of the three plasma circRNAs can be used as a diagnostic biomarker for hepatocellular carcinoma (AUC: .800) (Wu et al., 2020a); circ-KIAA1244 might represent a novel circulating diagnostic biomarker for gastric cancer (Tang et al., 2018) and circ-RPL15, a plasma circRNA, is a novel biomarker for chronic lymphocytic leukemia (Wu et al., 2020b). However, circRNAs have not received sufficient attention as circulating biomarkers for detection of BC. In the present study, we identified the significant differential expression of hsa_circ_0000091, hsa_circ_0067772, and hsa_circ_0000512 in plasma samples from BC patients, with these circRNAs subsequently used to establish a diagnostic model using the training set. The diagnostic model showed high specificity and sensitivity for BC detection and satisfactory stability in the test set. Moreover, the diagnostic model had a higher positive rate for BC detection than traditional circulating biomarkers (CEA, CA125, CA15-3, and CA19-9), suggesting that these results might describe a novel liquid biopsy method for diagnosing BC.

Tumor-derived circRNAs can be secreted into peripheral circulation and are useful for cancer detection (Li et al., 2015). Additionally, tumor-derived circRNAs play an important role in tumor progression and metastasis (Tkach and Théry, 2016); thus, we primarily focused on tumor-derived circRNAs. Using a circRNA microarray of BC tissue, followed by screening plasma samples and verification using both preoperative and postoperative plasma samples, we identified three differentially expressed circRNAs (hsa_circ_0000091, hsa_circ_0067772, and hsa_circ_0000512) from BC tumors. As expected, the diagnostic model using the three BC tumor–derived circRNAs showed improved specificity and sensitivity for diagnosing BC relative to traditional biomarkers.

Although the prognosis of BC patients has improved with the advent of precise treatment strategies, the incidence of BC metastasis continues to increase (Liang et al., 2020). A previous study reported that ∼20–30% of BC patients may develop metastases after diagnosis and primary tumor treatment, and ∼90% of cancer-related deaths are attributed to metastasis (Cancer Genome Atlas, 2012). Therefore, investigating effective biomarkers for BC prognosis is of considerable medical significance. Circulating RNA can be used not only as diagnostic markers but also prognostic markers for cancers (Hamam et al., 2017; Zaporozhchenko et al., 2018). For example, circulating miRNAs are used as blood-based markers for patients with primary and metastatic breast cancer (Roth et al., 2010). The present study found that the expression of hsa_circ_0000091 in BC plasma decreased again after metastasis, with the low expression of hsa_circ_0000091 correlating with poor prognosis in BC patients. These findings suggest that hsa_circ_0000091 could be a prognostic biomarker for BC.

Accurate assessment of ALN status in BC patients is important not only for choosing the right axillary treatment to avoid unnecessary axillary surgery and complications but also to save surgery time. Currently, sentinel lymph node biopsy (SLNB) and ALN dissection (ALND) are the main methods used to evaluate ALN status for BC patients during surgery. SLNB is less invasive than ALND and has become the gold standard for evaluating ALN status (Langer et al., 2007). Moreover, the complication rate of SLNB is significantly lower than that of ALND, although several complications, such as lymphedema, still occur in BC patients receiving SLNB treatment (Langer et al., 2007). Ultrasound has been widely used to assess ALN status before surgery (Cools-Lartigue and Meterissian, 2012), but reports suggest that the diagnostic capacity of axillary ultrasound is poor in estimating ALN metastasis (AUC: .585–.719) (Youk et al., 2017), which was similar to the present findings [AUC: .660 (95% CI: .565–.754)]. Analysis of the relationship between circRNAs and clinical features revealed that hsa_circ_0000091 expression in plasma was positively correlated with ALN metastasis and showed a high AUC [.728 (95% CI: .645–.811)] for identifying ALN metastasis. Notably, the AUC increased to .808 (95% CI: .739–.877) after using plasma hsa_circ_0000091 combined with ultrasound. Therefore, plasma hsa_circ_0000091 combined with ultrasound might offer the possibility of a minimally invasive method to determine the ALN status of BC patients before surgery, which could alleviate the complication rate by avoiding unnecessary axillary surgery.

The most common function of circRNAs is as a miRNA sponge (Tang et al., 2021). In the present study, investigating the downstream regulatory mechanisms of the identified circRNAs revealed that hsa_circ_0000091, hsa_circ_0067772, and hsa_circ_0000512 potentially regulate 82 target miRNAs, all of which participate in key biological signaling pathways. These results imply that hsa_circ_0000091, hsa_circ_0067772, and hsa_circ_0000512 possibly affect BC progression and metastasis through multiple signaling pathways, providing a theoretical basis for their use as markers. In future studies, we will explore the specific biological functions of these circRNAs *in vitro* and *in vivo* to obtain more definitive evidence.

## Conclusion

The present study established a BC diagnostic model using a combination of three tumor-derived plasma circRNAs (hsa_circ_0000091, hsa_circ_0067772, and hsa_circ_0000512), which potentially offers a valuable liquid biopsy method for diagnosing BC. Furthermore, the results indicated that the plasma hsa_circ_0000091 level might represent a prognostic biomarker for BC and that its combination with ultrasound can potentially serve as a new approach to predicting ALN metastasis.

## Data Availability

The original contributions presented in the study are included in the article/Supplementary Material; further inquiries can be directed to the corresponding author.

## Abbreviations

ALND, axillary lymph node dissection; ALN, axillary lymph node; AUC, area under the curve; BC, breast cancer; CA125, carbohydrate antigen 125; CA15-3, carbohydrate antigen 15-3; CA19-9, carbohydrate antigen 19-9; CEA, carcinoembryonic antigen; circRNA, circular RNA; FC, fold change; CI, confidence interval; DFS, disease-free survival; miRNA, microRNA; qRT-PCR, quantitative reverse transcription polymerase chain reaction; ROC, receiver operating characteristic; SD, standard deviation; SLNB, sentinel lymph node biopsy; VEGF, vascular endothelial growth factor; IL-3, interleukin 3.
